# Highly-Sensitive Refractive Index Sensing by Near-infrared Metatronic Nanocircuits

**DOI:** 10.1038/s41598-018-29623-z

**Published:** 2018-07-30

**Authors:** A. R. Rashed, B. Gudulluoglu, H. W. Yun, M. Habib, I. H. Boyaci, S. H. Hong, E. Ozbay, H. Caglayan

**Affiliations:** 1 Laboratory of Photonics, Tampere University of Technology, 33720 Tampere, Finland; 2 Nanotechnology Research Center, Bilkent University, Bilkent, 06800 Ankara, Turkey; 3 Hacettepe University, Nanoscience and Nanomedicine Department, 06800 Ankara, Turkey; 4 Components & Materials Research Laboratory, Electronics and Telecommunication Research Institute (ETRI), Daejeon, 305-350 Republic of Korea; 5 Hacettepe University, Food Engineering, 06800 Ankara, Turkey

## Abstract

In this work, we present a highly-sensitive refractive index sensor based on metatronic nanocircuits operating at near-infrared spectral range. The structure is designed based on simple nanorod geometry and fabricated by nanopatterning of transparent conducting oxides. The functionality of these polarization dependent metatronic nanocircuits is enhanced by applying tunable response. This feature is investigated by depositing NH_2_ (Amine) groups via plasma polymerization technique on top of indium-tin-oxide nanorods. The dielectric constant of Amine groups is a function of their thickness, which can be controlled by the RF power and the time duration of the applied plasma polymerization process. The resonance wavelengths of nanocircuits shift to higher wavelength, as the dielectric constant of the deposited material increases. An excellent agreement between the design and experimental results are obtained. Our metatronic based nanosensor offers a high-sensitive performance of 1587 nm/RIU with a satisfactory figure of merit for this class of sensors.

## Introduction

One of the promising photonic applications, by material design and geometrical engineering, is plasmonic based index sensing^1–4^. Due to the underlying quantum mechanical limitations, the transmittance resonance of near-infrared (NIR) range structures, usually are much broader and weaker than that of resonators operating in the visible range. For this reason, realizing high performance sensors in NIR range is much challenging. Even if plasmonic based sensors with high sensitivity and high figure-of-merit (FOM) are achievable in visible regime, still they suffer from their lossy performance, because of the high intrinsic optical absorption of the materials in this spectral range. On the other hand, not only attaining sharp and strong resonances in NIR range is difficult, but also, moderating the optical losses of those sensor devices remains as a significant challenge^5–8^.

Some efforts to design refractive index sensing devices based on plasmonic nanostructures could provide high values of sensitivity and FOM, but still these works are dealing with complicated fabrication processes and loss problem. For instance, Shih *et al*.^9^ has reported an index sensing device in NIR wavelength range based on nanoporous gold disks with acceptable sensitivity of 525.7 nm per refractive index unit (RIU). However, the quality factor parameter could be compromising, due to the wide resonance of their design. Recently, gold nanodisks with perfect absorption properties have been reported as plasmonic sensor in NIR range^10^. This sensor presents a high FOM of 87, but the reported sensitivity is around 400 nm/RIU. In another work, a plasmonic nanostructure named as gold mushroom arrays is fabricated by using interference lithography with a good sensitivity of 1015 nm/RIU and high FOM of 108^11^. However, the applied fabrication method is quite complicated, and not suitable for designing large-area sensing devices. In addition, the structure performance can be limited because of high absorption of the plasmonic materials in NIR range.

In order to investigate the refractive index correlated properties of a designed sensor, finding an effective method to tune the refractive index of the target material could be important. In one work, the refractive index of different polymers in visible wavelengths is tuned versus to the concentration of multiple developed nanosize organic dopants^12^. Tao *et al*. have investigated the preparation of polymeric nanocomposites with high refractive index by grafting a polymer chains onto the surfaces of TiO_2_ nanoparticles. They have shown that by increasing the volume fraction of the coupled polymer chains to the surface of TiO_2_, the refractive index dispersion of polymer-grafted TiO_2_ composites modifies^13^. The other technique is to utilize plasma copolymerization process to create modification of the refractive index. This technique has been exploited to fabricate thin films of two (or more) monomers. By modifying the ratio of the fed monomers, the chemical composition can be controlled to manipulate the optical properties of the deposited films^14^. In the other study, Zhang and coworkers have shown that how the thickness modification of the polymeric films changes their refractive indices. Such thickness modification was achieved by irradiating those polymeric films to UV light at different exposure times^15^.

Another recent focus of photonics is overcoming fabrication obstacles of sub-wavelength nanoscaled circuits. Applying materials with appropriate dispersion and absorption properties in the structure of nanocircuits provide the possibility to translate fundamental lumped circuit elements to optical frequencies^16,17^. Such tremendous achievement opens up a new avenue for functional applications of optical electronics^18–20^. Transparent conductive oxides as plasmonic materials with low-loss properties in the NIR regime^21–23^ are promising candidates to realize metamaterial-inspired nanocircuits (metatronics) and to integrate them with silicon-based photonic applications.

**Figure 1:**
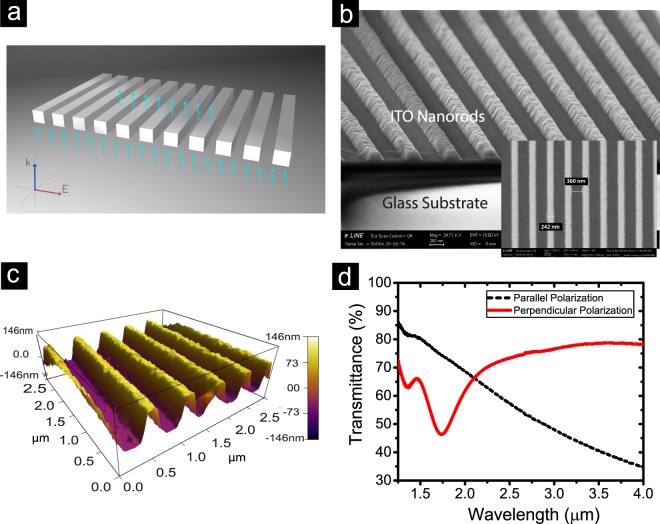
Design and characterization of the fabricated nanocircuits. (**a**) Schematic view (**b**) SEM image (**c**) 3D view generated by AFM measurement of the fabricated ITO metatronic nanocircuits. (**d**) Transmission spectra of patterned NRs in both parallel and perpendicular regimes of the incident E-field wave vector.

In this work, we apply optical nanocircuits made of rectangular shape nanorods (NRs) separated by the air gaps for high sensitive refractive index sensing applications in NIR spectral range. An optimized design of such NRs (scheme in Fig. 1(a)) are fabricated by applying nanoimprint lithography and inductively coupled plasma (ICP) etching processes on the indium-tin-oxide (ITO) thin films. Such arrangements of plasmonic (with negative real part of permittivity) and dielectric (with positive real part of permittivity) materials lead to band-pass and band-stop filters in optical frequencies^24,25^. The polarization dependent response of nanocircuits can be exploited to enhance their performance for sensing applications. The realized nanocapacitors in the structure can be changed due to the applied surface modification, which results in resonance shift. Utilizing ITO as a plasmonic material^26–28^ to fabricate the building blocks of the designed device provides a low-loss sensor in NIR regime, which makes our design distinguishable in comparison to other designs. Moreover, the applied fabrication method of nanoimprint lithography is the key point to produce low-cost sensor devices with large-areas. Furthermore, we present a simple method to produce a tunable high refractive index material based on plasma polymerization (PlsP) process. We show that by controlling the polymerization process parameters such as the exposure time and the radio frequency (RF) power we can achieve refractive index modification of deposited Amine groups as their thicknesses change. Quantitatively and qualitatively, an excellent agreement between the obtained numerical simulations and the experimental results are observed. We do believe that our realized metatronic based nanosensor device is a promising candidate for developing low-cost bulk refractive index sensors in NIR range with high sensitivity on a large scale.

## Results

### Structural and optical characteristics of nanocircuits

The low-loss plasmonic properties of ITO in NIR range makes it a promising candidate to fabricate the designed refractive index sensor. The ellipsometry characterization results on 145 nm of ITO deposited on glass substrate show the plasmonic behavior beyond its epsilon-near-zero (ENZ) wavelength (see Fig. S1). Figure 1(b) and (c) show the scaning electron microscopy (SEM) image and generated 3D view by atomic force microscopy (AFM) image of the fabricated ITO NRs array, respectively. The optical response of ITO NRs can be tailored by varying width (w) and height (h) of ITO rods, air gaps width (g) and the polarization state of the incident light. The applied master template with a period (w + g) of 600 nm results in NRs array with geometries of w = 240 nm, g = 360 nm and h = 145 nm, confirmed by AFM results. As it is presented in Fig. 1(d), the optical responses of these NRs are dependent on the polarization of the E-field of the incident plane wave, resulted from their metatronic properties^29^. Each component of plasmonic ITO NR functions as a nanoinductor parallel with a nanoresistor, while the dielectric air gap region acts as a lossless nanocapacitor. The perpendicular polarization of the E-field vector with respect to the rods orientation results in functionality of NRs arrays as band-stop filters, while in parallel polarization regime the arrays act as band-pass filters (Fig. 1(d))^24^.

In perpendicular polarization of the incident wave, one can tune the spectral position of band-stop region by varying NR geometries. Caglayan *et al*. showed that lower possible values of w/g accompanied with deeper air gaps of patterned ITO shift transmittance resonances of the structure towards ENZ wavelength of the applied ITO^24^. Take it into account that the lower positive values of the imaginary part of the dielectric constant for ITO close to ENZ wavelength guaranties the higher quality factor of the transmission resonances^30^. This is a necessary condition to realize a highly-sensitive plasmonic sensor with a satisfactory high FOM, since this parameter has a direct relation with the quality factor of the resonator^31^. Details of the optimization process of the designed template to pattern ITO films are discussed in section II of the Supplementary Information (Fig. S2).

### Modification of nanocircuits surface properties by PlsP process

We used the PlsP technique to modify the optical properties of the ITO NRs by depositing Amine groups on their surfaces. By adjusting the RF power and the time duration of the applied PlsP process, one can control the thickness of deposited material. The increase of the RF power results in the enhancement of ionized gas amount and plasma density. Therefore, the efficiency of polymerization process enhances. While by increasing the exposure time, the interaction between the sample surface and produced plasma enhances. Consider that after a particular condition, increasing these two PlsP process parameters will not result anymore in growing the thickness of deposited monomers with a linear rate^32,33^. The AFM images presented in Fig. S3 show that how the surface of a bare glass alters after growing Amine groups on it. Our investigations show that by the physical deposition of the Amine groups on top of ITO or glass substrates, there is no chemical or optical interaction between NH_2_ molecules and these substrates. Furthermore, the constant absorption trend of Amine groups in the resonating range of ITO NRs approves this fact that their introduced weak absorption does not play any role in reshaping or tuning of the ITO NRs resonances.

**Figure 2:**
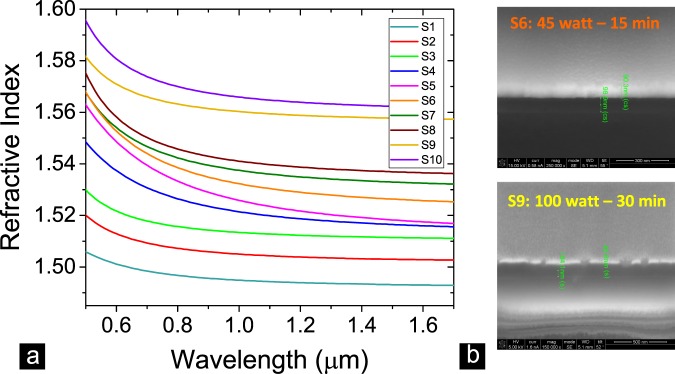
Refractive index modification of Amine groups versus their thicknesses. (**a**) The measured refractive indices of deposited Amine monomers on glass substrates by ellipsometry method. These results show that we can tune the refractive indices of polymerized Amine monomers by controlling parameters of the PlsP process. **(b**) Cross-section views of S6 and S8 reference samples acquired by FIB method to measure the thicknesses of deposited Amine groups. These samples were coated with 100 nm and 50 nm of gold, respectively, to prevent charging effect on the surface of the samples. These check measurements are done to approve the correctness of the extracted results by ellipsometry method. The achieved results based on both methods are relatively in good agreement.

For investigating the physical and optical properties of the Amine groups, we prepared ten control samples by depositing them on bare glass substrates. In parallel to that, we prepared ten ITO NRs coated with Amine monomers as main samples. We used the ellipsometry technique to acquire the thicknesses and the corresponding refractive index of grown Amine groups on the surface of control samples (see Table 1). Figure 2(a) clearly shows the association of the refractive index to the thickness of deposited Amine groups. These results prove that by growing Amine groups on a particular surface the refractive index parameter is enhanced. We acquired the cross-sectional view of some control samples by focused ion beam (FIB) method to justify the correctness of the acquired thicknesses (Fig. 2(b)).

**Table 1:** The extracted refractive indices in NIR spectral range and thicknesses of control samples prepared at ten different conditions of the plasma polymerization process based on ellipsometry method.

Sample Label	Applied Plasma Conditions	Refractive Indices of Amine Groups in NIR Range	Amine Groups Thicknesses (nm) (Ellipsometry Data)
S1	05 Watt - 05 min	1.4931	6.2
S2	10 Watt - 05 min	1.5034	12.7
S3	15 Watt - 05 min	1.5118	18.5
S4	15 Watt - 10 min	1.5175	61
S5	15 Watt - 15 min	1.5199	86.2
S6	45 Watt - 15 min	1.5276	104.2
S7	75 Watt - 15 min	1.5339	127.6
S8	100 Watt - 15 min	1.5378	131.4
S9	100 Watt – 30 min	1.5582	161.7
S10	100 Watt - 45 min	1.5631	224.5

Moreover, the performed AFM analyses on most of the control samples confirm the correctness of the thickness results extracted by ellipsometry method. The acquired results based on both methods are relatively in a good agreement with an average deviation error of 5%. Accordingly, this technique can be used to modify surface properties of nanocircuits in order to investigate their potential application as an index sensing device.

Fourier transform infrared (FTIR) spectroscopy performed on different Amine coated samples reveals that the plasma reactions induced the formation of various components, such as aromatic and aliphatic N-H, carbonitride and hydrocarbon components (See Supplementary Information section IV). These components experience apparent modifications during the PlsP process as the exposure time or the RF power alters. Such modification in chemical components of the deposited Amine could be the reason for the modification of the refractive index versus their thickness change^14,34–36^.

### Refractive index sensing features of nanocircuits

**Figure 3:**
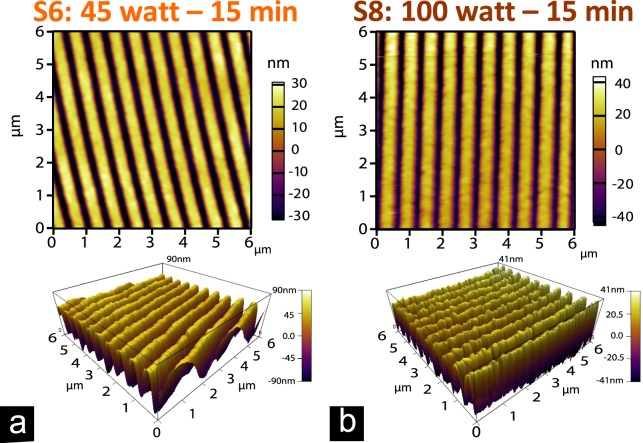
Surface modifications of ITO NRs. AFM images related to the surface of two identical ITO NRs after plasma polymerization process by Amine groups in two different conditions of (**a**) 45 watt-15 min (**b**) 100 watt-15 min. The modification of samples surfaces is obvious in 3D views of both samples versus to the bare sample surface presented in Fig. 1(c).

The applied treatments on the surface of ITO NRs by PlsP technique allow us to realize the tunable feature of the designed structures. Figure 3 presents the AFM results of two identical ITO NRs coated with Amine monomers under 45 watt and 100 watt of the input RF power, while for both samples the exposure time to glow discharge is considered as 15 minutes. The surface modification is evident as compared to the image of bare NR presented in Fig. 1(c). The provided 3D views of these samples are a good proof for the growth of Amine monomers on top of the nanostructures, in which higher applied power value resulted in more filling of the air gaps and higher roughness of the sample surface.

**Figure 4:**
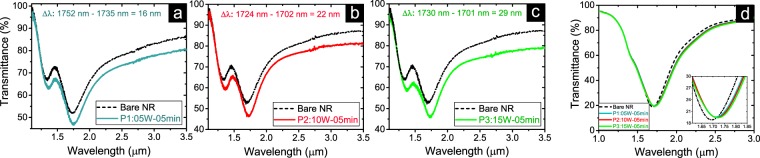
Evaluating sensing performance of Amine coated nanocircuits with respect to their corresponding bare structures. The exposure time to glow discharge is kept constant as 5 minutes, while the input power is changed as (**a**) 5 watt, (**b**) 10 watt and (**c**) 15 watt. The amount of the resonance wavelength shifts of coated NRs with respect to that of bare NRs are presented on each figure. (**d**) Simulation results for the observed red-shift of the resonance band for three presented cases in parts (**a**) to (**c**). The inset shows a closer view of the resonance shifts.

**Figure 5:**
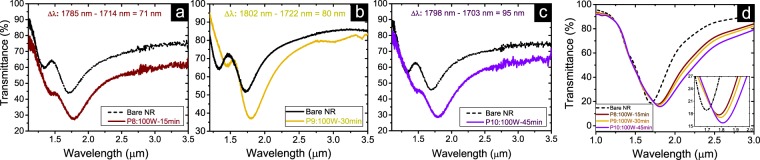
Evaluating sensing performance of Amine coated nanocircuits with respect to their corresponding bare structures. The input power is kept constant as 100 watt, while the exposure time to PlsP process is modified as (**a**) 15 minutes, (**b**) 30 minutes and (**c**) 45 minutes. The amount of the resonance wavelength shifts of coated NRs with respect to that of bare NRs are presented on each figure. (**d**) Simulation results for the observed red-shift of the resonance band for three presented cases in parts (**a**) to (**c**). The inset shows a closer view of the resonance shifts.

Figures 4 and 5 present the resonance wavelength modification of ITO NRs due to applied surface treatments by depositing Amine groups in the air gaps and top sides of the nanocircuits. In Fig. 4, the transmittance of three ITO NRs coated with Amine monomers in three different PlsP process conditions are compared to their resonance bands before deposition process. During the polymerization processes of these samples the RF power is adjusted as 5, 10 and 15 watt, respectively, while the exposure time is kept constant as 5 minutes. The tuning of the resonance wavelength is evident for all three samples, in which for higher input power values of the applied PlsP process, further red-shifts of transmission resonances is observed. The acquired shift values for these three samples increases linearly as a function of refractive indices of deposited Amines. This occurs since for the applied PlsP conditions, the achieved refractive index of deposited Amines increases almost linearly, while the thickness parameter does not alter so much. As a support for the presented experimental results, Fig. 4(d) shows the simulated resonance wavelength shifts corresponding for the observed red-shift for each one of the samples. Figure S5 presents the experimental and simulation results for the other group of samples in which their surfaces are exposed to glow discharge for 15 minutes, while the input power parameter is applied as 15, 45 and 75 watt. According to higher achieved refractive indices for thicker deposited layers of Amine monomers, further red-shift of resonance wavelengths are obtained with respect to that of the samples presented in Fig. 4. The rate of the red-shift for the samples presented in Fig. S5 does not change drastically with respect to that of the samples presented in Fig. 4. This occurs even if the thicknesses of Amine groups grows much faster for S5 to S7 than S_1_ to S_3_ samples, while their refractive index increases with the same rate of those samples (see Table 1). One can conclude that at higher thickness values of deposited Amine groups, the observed resonance shift values influenced by the refractive index change, rather than the Amine thickness change.

The red-shift of the resonance band related to three similar ITO NRs after coating with different thicknesses of Amine monomers is presented in Fig. 5(a–c). The exposure time of samples is adjusted as 15, 30 and 45 minutes, while the same RF power of 100 watt is applied. Increasing the time duration of the plasma process results in further red-shift of the nanocircuits resonance wavelengths. In Fig. 5(d), the simulation results associated to the observed red-shifts of nanocircuits resonances are shown. Figure S6 presents the experimental and simulation results for the other group of samples in which the RF power parameter is kept constant (15 watt) for all samples, while the time duration of the polymerization process is considered as 5, 10 and 15 minutes. A similar trend for the modification of the resonance is observed for these samples. However, since the time and power parameters are considered in lower values with respect to the applied PlsP conditions for samples presented in Fig. 5, the less amount of red-shift is expected.

**Figure 6:**
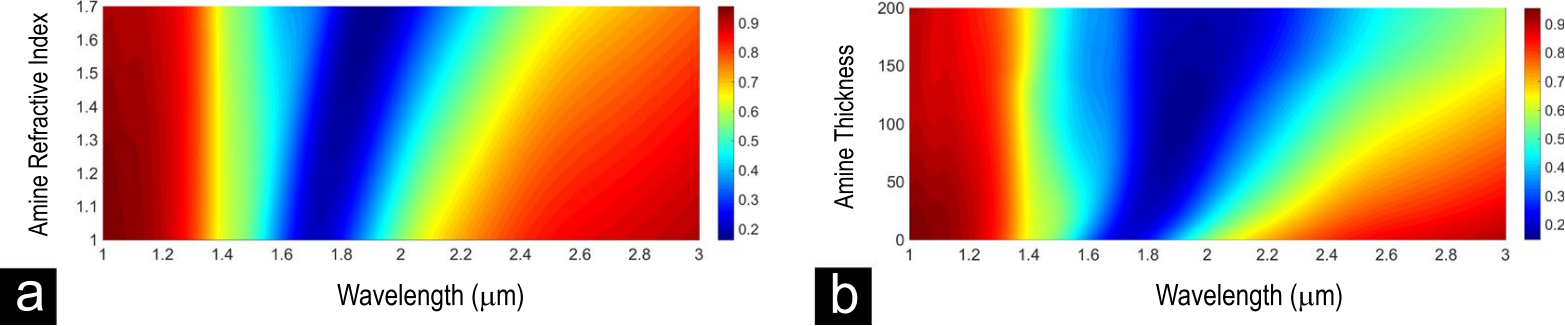
ITO NRs resonance modification versus thickness and refractive index of the target material. The modification of the ITO NRs resonance spectrum versus (**a**) the refractive index change of the deposited material on top of the nanocircuit from 1 to 1.7, while the thickness parameter is kept constant as 50 nm. (**b**) the thickness change of the deposited material on top of the nanocircuit from 0 to 200 nm, while the refractive index parameter is kept constant as 1.5.

Overall, the presented red-shifts in the resonance wavelengths of the nanocircuits is correlated to both refractive index and thickness parameters of the deposited material. Finite difference time domain (FDTD) simulation results in Fig. 6(a) show that for a constant thickness value of the deposited material on the surface of NRs, the increase of the refractive index shifts the structure resonance to higher wavelengths. This occurs because of the dielectric constant alteration and consequently the capacitance modification in the air gaps^24^. Moreover, the presence of the material on top sides of NRs creates an additional capacitance in series with RL circuit standing for ITO rods, resulting in further red-shift of the transmittance resonance band^37^.

By increasing the thickness of the deposited material on top of nanocircuits, while the refractive index parameter is kept constant, the transmission resonance shifts slightly to higher wavelengths (Fig. 6(b)). This occurs by modifying the effective dielectric constants of the capacitances, appeared in the air gaps and on top of ITO regions. However, above specific thicknesses of the deposited material (such as 50 nm) the broadening and reshaping of the resonance occurs rather than the red-shift effect. The broadening effect is apparent in both experimental and simulation results presented in Figs 5 and S6 at higher thicknesses of deposited material. Note that in Fig. 6(a) the broadening effect is not pronounced as much as red-shift effect. Thus, as it is aforementioned, the observed red-shift of the NRs resonances originate mostly from the refractive index modification rather than thickness modification of deposited material. All these effects can be investigated more in details by modeling the nanocircuits based on circuit theory as a future study.

**Figure 7:**
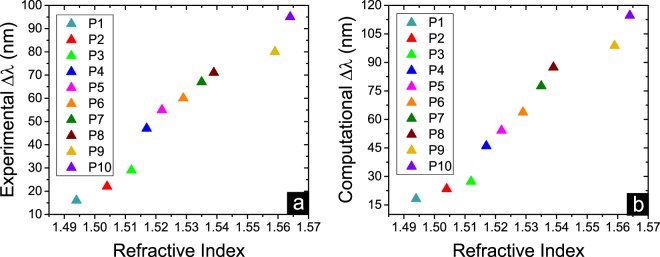
Simulating sensing results by FDTD method. (**a**) Experimentally measured and (**b**) Simulated red-shift values of the designed sensor transmittance band for different values of the target material refractive index. There is a good agreement between the experimental and simulation results.

The experimental and simulation red-shift values of the nanocircuits resonances resulted from different refractive indices of deposited material are summarized in Fig. 7. In addition, the primary calculated index sensing results based on circuit theory is presented in section VI of Supplementary Information (Fig. S7(a)). It is evident that for all samples increasing the input power and the time duration of the polymerization process tunes the resonance to longer wavelengths. Interestingly, both experimental and simulation results are following the same trend which is the proof of functionally good agreement between these results.

The achieved good red-shift values corresponded to the refractive index changes of the material positioned on the surface of ITO NRs allows us to exploit these structures for the sensing applications. We can eliminate the effect of material thickness on the performance of the designed refractive index sensor by keeping it equal for the studied materials. A standard sensor evaluation method is used for calibration and to determine the detection limit of the sensor device. Corresponding refractive index change is recorded as a shift in the transmission spectrum. A red-shift in resonance wavelength occurred when the refractive index of deposited material on top of the structure is changed. The quality factor of the resonance band slightly drops after positioning the target material on top of the device. However, the broadening effect can be neglected at lower thicknesses of the deposited material. By considering that the effect of deposited Amine thickness on the red-shift of NRs resonances is negligible after 50 nm thickness value, we calculated the sensitivity for the last six samples. By taking the average, we report the sensitivity of our device as ~1587 nm per RIU.

Figure of Merit (FOM) is another key parameter to evaluate the performance of a sensor device. This parameter is defined as (Δλ/Δn)(1/Δω) where Δλ is the wavelength shift, Δn is the refractive index modification and Δω is the full-width of the resonant dip at half-maximum^31^. The FOM determines the sensitivity of the sensor to measure very small wavelength changes of the resonance wavelength^38^. The average value for Δω of designed sensor device while it is deposited with Amine groups under PlsP condition is about 260 nm which results the FOM factor of 6.1.

## Discussion

In summary, we have designed and fabricated metatronic nanocircuits for refractive index sensing in NIR range. The applied nanoimprint lithography provides us the possibility to produce sample devices with large area. By utilizing ITO as the main material of our design we could achieve a low-loss, highly sensitive sensor devices, operating in NIR spectral range.

A high sensitivity of 1587 nm/RIU is achieved with our sensor device which is higher than most of the designed sensors operating in visible and NIR spectral ranges. Our design has an acceptable figure of merit (FOM) of 6.1 for this class of samples. The nanocapacitor values and correspondingly, the resonance wavelength of the sensor device change, as the dielectric constant of the nanoscale gap regions and ITO surface change. This can be achieved by modifying the surface of nanocircuits via depositing Amine groups on top of the device via PlsP technique. The dielectric constant of Amine groups is tuned a function of their thickness which is controlled by the RF power and the exposure time of the applied PlsP process. We show that this technique can be applied as a promising method to produce high refractive index material. This novel approach of realizing low-loss index sensing will open up new avenues for future research and practical applications. Our low-expense device is a promising method to realize ultra-high resolution biosensors, for detecting targets such as proteins, viruses and/or bacteria.

## Methods

### Nanofabrication processes of samples

ITO NR arrays were fabricated by applying nanoimprint lithography and inductively coupled plasma (ICP) etching processes. Commercial 150 nm thick ITO coated glass was purchased, and cleaned with IPA solution for 10 min. Then, thermal imprint resist (PBMA 7 wt% in chlorobenzene solution) was spin-coated at 3000 rpm for 1 minute on the ITO coated glass. It was covered by a master template which was structured to imprint the NR array. The used template to fabricate the samples has a period (w + g) of 600 nm. A nanoimprint tool was used to heat the stack of master template and substrate to a temperature of 140 °C and subsequently press it into the substrate at 20 bar for 20 minutes. The stack was cooled to room temperature and de-molded. An oxygen descum process was employed by RIE (reactive ion etching) equipment with 150 watt power and 40 mTorr pressure to remove any residual resist layer and control the ITO NR width. Then 30 nm Cr layer was deposited at 0.5 Å/s on the sample by e-beam evaporation. The resist was removed by lift-off in acetone sonication for 10 minutes leaving nanopatterned Cr on the ITO substrate. The ITO layer was patterned using an inductively coupled plasma reactive ion etch tool with a BCl_3_/Ar chemistry and 100 watt of RF power and 500 watt of ICP power employing the Cr layer as a hard mask. After etching process, the Cr layer was removed by dipping in commercial Cr etchant leaving the ITO NRs fabricated over large area (30 mm × 30 mm).

### Optical and structural characterization

Normalized transmission spectra were obtained in the spectral range of 1 µm to 3 µm using Vertex 70 series FTIR spectrometer (provided by Bruker) integrated with Hyperion IR microscope system. Using the microscope incoming light is focused on the patterned structures and ITO etched glass substrate which is used as the reference sample to acquire the background signal. The polarization state of the impinging light to samples is controlled by applying NIR-MIR polarizer in transmitted beam path. SEM images were obtained using a Raith E-Line SEM operating at 10 kV. AFM images of Bare and Amine coated ITO NRs are acquired by using a commercial atomic force microscopy (MFP-30, Asylum Research) setup. Variable-angle high-resolution spectroscopic ellipsometry (J. A. Woollam, V-VASE) was used to determine the thicknesses and optical constants of the Amine coated thin films. The cross-section views of Amine coated reference samples are acquired by Nova 600 NanoLab Dual Beam SEM/FIB instrument provided by FEI company.

### Plasma polymerization technique

Glow discharge plasma polymerization technique is used for the surface modification of the ITO NRs to create Amine groups. For this purpose, Allylamine (AA) was used as a precursor. The glow discharge treatments were carried out in RF (13.56 MHz) Plasma Polymerization System (Diener electronic GmbH + Co. KG, Germany). The plasma system consisted of a vacuum chamber with two inlets having pressure gauges and frequency power components with the RF and electrodes wrapped around the vacuum chamber. The monomer tank is connected to the reactor through a flowmeter and a needle valve. The samples were placed on to the ground electrode in the middle of the reactor and the reactor was evacuated to 10^−1^ mbar pressure. In order to prevent impurities, the monomer tank was chilled with liquid nitrogen and the degassing was performed until the reactor inner pressure reached to 10^−1^ mbar. After 10 minutes continued vacuum procedure, monomer valve was opened; defrosted amine monomer vapor is allowed to flow though the reactor at a flow rate of 35 ml/min to remove the remaining air in the chamber. Then, RF power was adjusted in different values between 5 to 100 watt and the ITO NRs surfaces were exposed to glow discharge for different time values between 5 to 45 minutes. Power losses were minimized by means of a matching network. At the end of the process, the RF generator was turned off and the system was fed with argon gas for 10-15 minutes to deactivate free radicals.

### Simulations

The optical properties of the sensor device have been simulated by means of a FDTD method, implemented with the aid of a commercial software package (Lumerical FDTD Solutions). For the calculation of normalized transmittance of Bare ITO NRs, the extracted dimensions by AFM method have been used in the software. The optical constants of ITO and Amine groups have been considered based on the extracted results by ellipsometry method. Unit cell of the design have periodic boundaries along x and y-axes, while the perfectly matched layer (PML) in the z-axis. The injection of the source is performed in the z (propagation) direction with a normal incident and transmitted signal collected in other side of the structure.

## Electronic supplementary material

Supplementary Information
