# Supplementary material for: Highly-Sensitive Refractive Index Sensing by Near-infrared Metatronic Nanocircuits
Source: Sci Rep. 2018 Jul 30;8:11457. doi: 10.1038/s41598-018-29623-z (PMC6065432; doi:10.1038/s41598-018-29623-z)
Supplement: Supplementary file 1 — Supplementary Information [file 41598_2018_29623_MOESM1_ESM.docx]

**Supplementary Information for:**

**Highly-Sensitivity Refractive Index Sensing by Near-infrared Metatronic Nanocircuits**

A. R. Rashed^1.2,*^, B. Gudulluoglu^3^, H. W. Yun ^4^, M. Habib^2^, I. H. Boyaci^5^, S. H. Hong^4^, E. Ozbay^2^ and H. Caglayan^1,*^

^1^Laboratory of Photonics, Tampere University of Technology, 33720, Tampere, Finland

^2^Nanotechnology Research Center, Bilkent University, Bilkent, 06800, Ankara, Turkey

^3^Hacettepe University, Nanoscience and Nanomedicine Department, 06800, Ankara, Turkey

^4^Components & Materials Research Laboratory, Electronics and Telecommunication Research Institute (ETRI), Daejeon 305-350, Republic of Korea.

^5^Hacettepe University, Food Engineering, 06800, Ankara, Turkey

email: [alireza.rashed@tut.fi](mailto:alireza.rashed@tut.fi), [humeyra.caglayan@tut.fi](mailto:humeyra.caglayan@tut.fi)

1. **Optical Characteristics of the ITO Film**

Figure S1 shows the experimental results achieved by ellipsometry method for the refractive index and extinction coefficient of the ITO, used for fabricating the ITO NRs. The commercially available ITO wafers on glass substrate has the thickness around 150 nm. Our ellipsometry on un-patterned samples and AFM measurements on ITO NRs identify the thickness value of 145 nm which is close to reported value by provided company. In perpendicularly polarized transmission spectrum of ITO NRs, an unpronounced resonance around ENZ wavelength (1.33 μm) of un-patterned ITO appears. This is a clear evidence of occurring a Berreman mode in the ENZ region of the un-patterned ITO, in which appears in p-polarization mode of the incident electromagnetic wave.


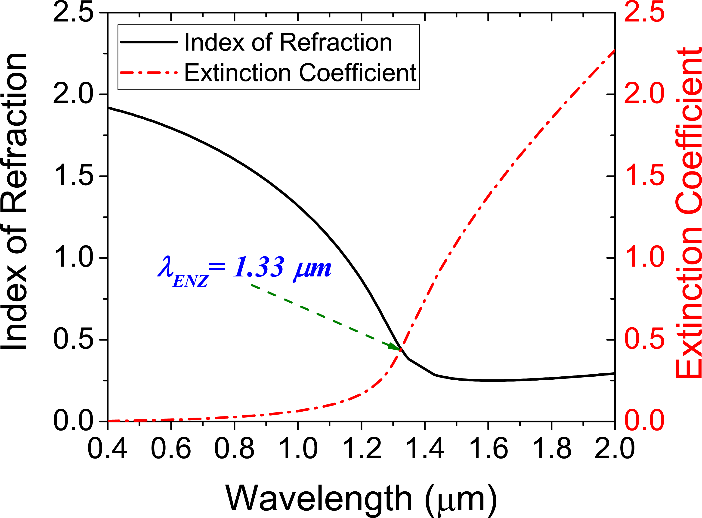


**Figure S1.** **Optical properties of ITO film used to fabricate sensors.** The measured refractive index and extinction coefficient of the ITO film on glass substrate. The ENZ wavelength of the annealed sample is appeared at 1.33 μm.

1. **Optimization procedure of metatronic based ITO NRs**

The master template to print ITO NRs is designed based on the performed computations on the simulated model of the sample. To do this, we swept all three parameters of height, width and period of the structure versus to each other. Our simulation results show that for a constant period of the NRs array, the increasing NRs width decreases the quality factor of the structure and red-shifts smoothly the resonance band. However, by considering the fabrication limitations and desired optical properties, the optimum value of 240 nm for the NRs width is decided, while the period is fixed as 600 nm (Fig. S2(a)). The presented simulation results in Fig. S2(b) show that decreasing period for the fixed ITO width and height values results in broadening of the resonance band, which reaches to our fabrications limits. However, after a specific value (in this design 600 nm) the resonance band position and bandwidth remain almost the same. Thus, the period of master template is considered as 600 nm. Fig. 2(c) shows the influence of NRs height on the transmission resonance. For higher heights of NRs, we observe more blue-shift of the resonance band towards the ENZ wavelength of the ITO which guaranties lower absorptive loss for the structure even if a slight broadening of the resonance band is occurring. Therefore, the deepest possible air gaps of NRs arrays which for our case is 145 nm is decided.


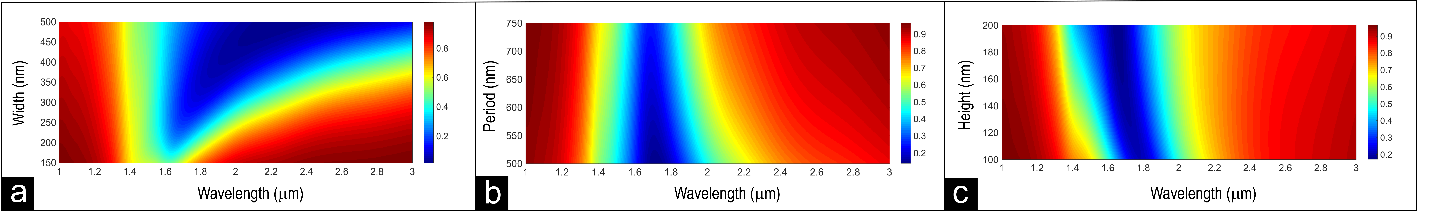


**Figure S2.** **Optimizing process of ITO NRs for sensing purposes.** The numerical simulation for the transmission spectrum of the nanocircuits resonance band versus (**a**) width (**b**) period and (**c**) height of ITO NRs to design a proper master template used in nanoimprinting process.

1. **Modification of Bare substrates surfaces by depositing Amine groups**

Figure S3(a) and (b) show the AFM images of a bare ITO and glass on glass substrate, respectively. The rough surface of the ITO sample is evident as compared to the bare glass sample surface. Such topography of the ITO surface affects the roughness of the Amine groups which are deposited on the ITO surface. The expected pronounced roughness of the Amine surfaces is taken into account in the simulated model of Amine coated ITO NRs. Figure S3(c) shows a 3D image of the Amine coated glass substrate, extracted from the AFM measurements. The applied modification by the PlsP process on the surface of the glass substrate is apparent by comparing the presented AFM images. This is a proof for the treated surface of the substrate after exposing to glow discharge.


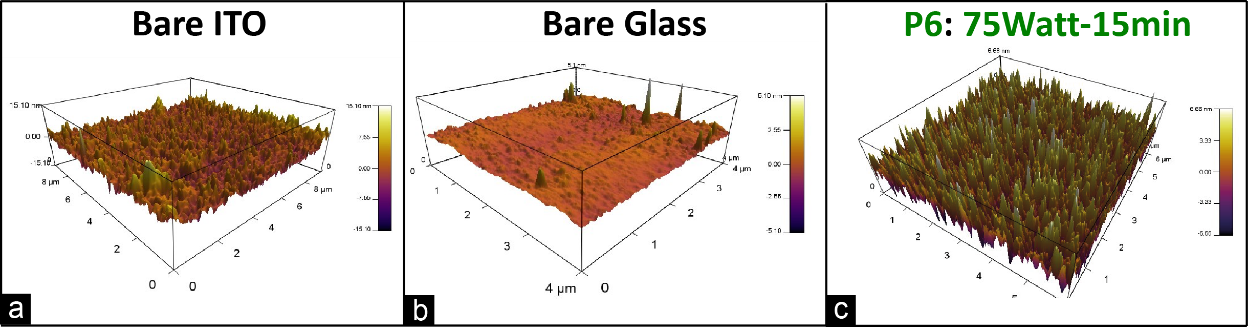


**Figure S3.** **Substrates surface modification after Amine groups deposition.** The 3D images (extracted from the AFM measurements) of (**a**) bare ITO surface, (**b**) bare glass surface and (**c**) Amine coated glass substrate by exposing the sample for 15 minutes to glow discharge under applying 75 watt of RF power.

1. **Investigation of the correlation between refractive indices of the Amine groups and their thicknesses**

As it is evident from Fig. S4, by modifying RF power (Fig. S4(a)) or exposure time (Fig. S4(b)) parameters, while the other parameter is kept constant, some particular peaks corresponding to different chemical components are enhanced. In the presented FTIR spectrum of the deposited Amine films the peaks in the 3600-3220 cm^-1^ region, such as 3355 and 3220 cm^-1^ are correspond to asymmetrical and symmetrical stretching N-H groups, respectively. In addition, this region could be included OH groups. The observed peaks in the 3000-2750 cm^-1^ range are indicating the aliphatic ν(C-H) stretching modes. The peaks in the region of 2250-2100 cm^-1^ are related to stretched ν(C≡N) groups. The presented peak in 1643 cm^-1^ is related to primmer Amine in-plane bending. The peak in 1456 and 1381 cm^-1^ are corresponding to bending δ_s_(C-H) and wagging ω(C-H) vibrations, respectively. The out-of-plane aromatic C-H deformation (γ) at 754 cm^-1^ and aromatic ring (C=C) rocking motion (δ) at 699 cm^-1^ can be observed. The modifications of these modes at each new applied PlsP condition influence the chemical components of the deposited Amine and correspondingly its index of refraction^14^.


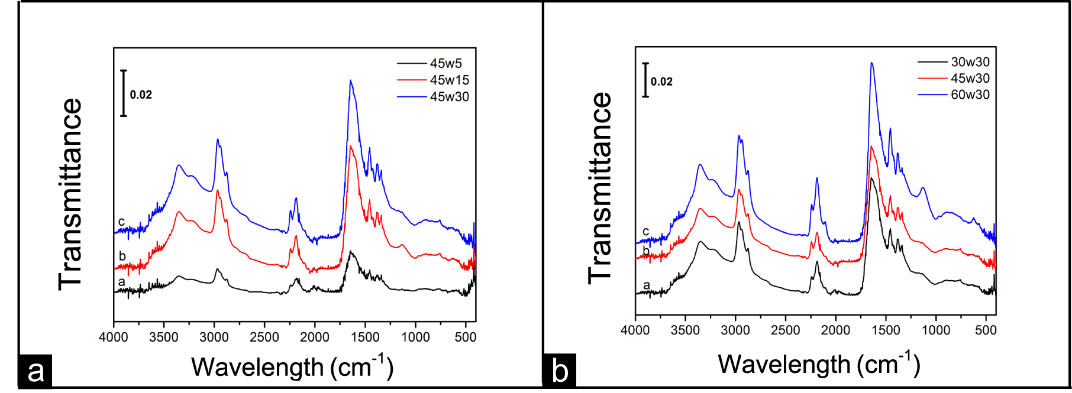


**Figure S4.** **Exploring the refractive index modification of Amine groups as their thicknesses change.** The FTIR spectroscopy results in MIR range for Amine coated samples (**a**) the RF power is considered constant as 45 watt, while the exposure time of PlsP is varied (**b**) the exposure time is considered constant as 30 minutes, while the RF power is varied.

1. **Refractive index sensing features of other groups of Amine coated nanocircuits**

Figure S5(a-c) presents the experimental results regarding the acquired red-shift of the resonance bands of nanocircuits versus different refractive indices of the deposited Amine monomers. In these experiments in order to control the refractive index of the polymerized Amine groups, the input power is increased as 15, 45 and 75 watt, while the time duration of PlsP process is kept constant as 15 minutes. By increasing applied time duration of the process, higher thicknesses and consequently refractive indices of deposited material expected. This results in realizing new position of the resonance band in higher wavelengths. However, since the applied parameters are in lower rates than the used conditions in experiments related to Fig. 4, lower red-shift values were acquired. Computational results presented in Fig. S5(d) are relatively in good agreement with the acquired experimental results.


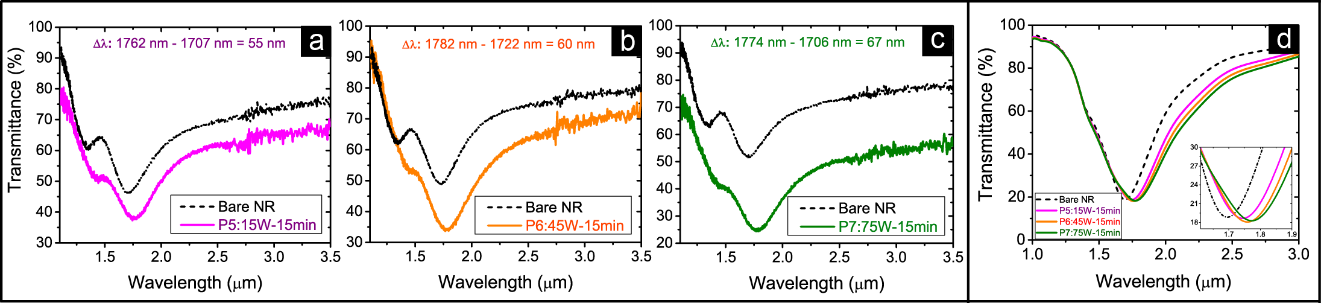


**Figure S5.** **Evaluating sensing performance of Amine coated nanocircuits with respect to their corresponding bare structures.** The time duration of the PlsP process is kept constant as 15 minutes, while the input power is changed as (**a**) 15 watt, (**b**) 45 watt and (**c**) 75 watt. (**d**) Simulation results for the observed red-shift of the resonance band for three presented cases in parts (a) to (c). The inset shows a closer view of the resonance shifts.

Figure S6 shows the experimental and computational results for another group of the samples in which power parameter is kept constant, while the time duration of the PlsP process is modified. Based on ellipsometry results, longer PlsP process results in higher refractive indices of the deposited Amine groups. Subsequently, more red-shift is observed in the resonance band of our plasmonic sensor as the time duration of the process is increased. Obviously, as a result of higher achieved refractive indices, the resonance band of nanocircuits shifts to longer wavelengths than that of those samples presented in Fig. 5. Figure S6(d) provides the simulation support for the experimental observations shown in (a) to (c) parts of this figure.


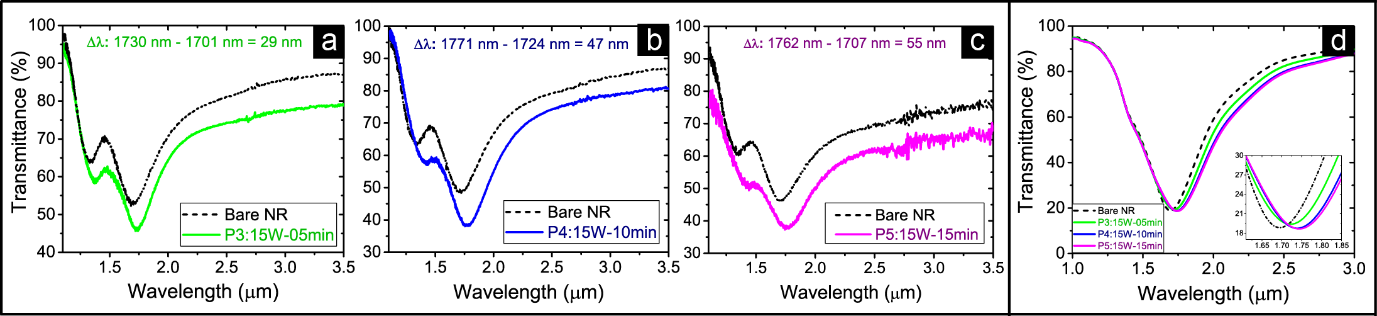


**Figure S6.** **Evaluating sensing performance of Amine coated nanocircuits with respect to their corresponding bare structures.** The input power is kept constant as 15 watt, while the time duration of the PlsP process is modified as (**a**) 5 minutes, (**b**) 10 minutes and (**c**) 15 minutes. (**d**) Simulation results for the observed red-shift of the resonance band for three presented cases in parts (a) to (c). The inset shows a closer view of the resonance shifts.

1. **Computational evaluation of the sensor performance based on Circuit Theory**

The performance of the ITO NRs can be analyzed based on circuit based theory as presented in reference 24. In such case, we model the designed nanostructures as lumped circuit elements. The air gap areas -filled with corresponding index and thickness from Table 1- are modeled as capacitor, while the NRs act as inductance and resistance. Depending on the electric field polarization of the incident plane wave, the modeled nanoinductor (with nanoresistor) and nanocapacitor form a series or parallel combinations to realize band-stop or band-pass filters, respectively. However, by considering the presence of a material like Amine monomers on the ITO NR surface the corresponding electric circuit of the structure will be much complicated. We consider this concept as the future work to perform a comprehensive study on the sensing performance of the nanocircuits based on circuit theory. However, our primary results show qualitatively a good agreement with the achieved experimental results. This is evident by comparing the presented results in Fig. S7(b) to the experimental results in Fig. S7(a).


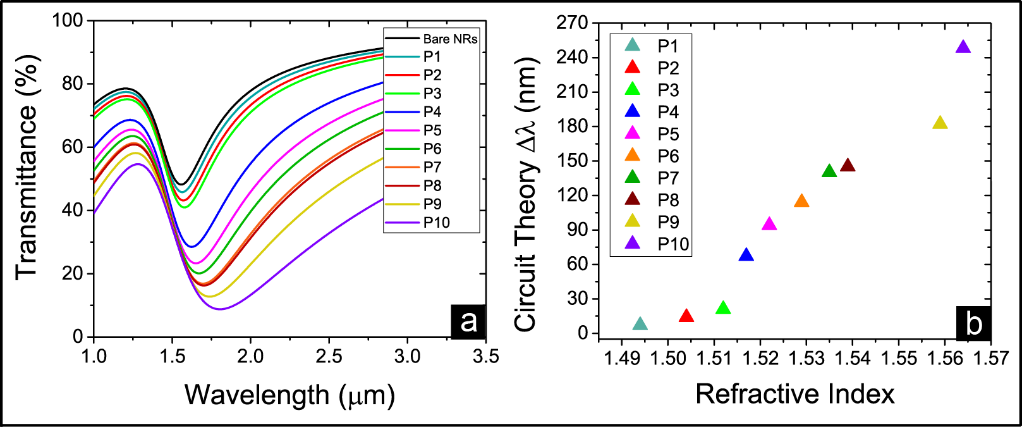


**Figure S7.** **Index sensing results based on circuit theory.** (**a**) Calculated transmittance of Amine coated NRs for different plasma coating conditions. (**b**) Calculated red-shift values of the designed sensor resonance band for different values of the target material refractive index. Qualitatively, there is a good agreement between these results with experimental ones.
